# Maternal Diet during Pregnancy Induces Gene Expression and DNA Methylation Changes in Fetal Tissues in Sheep

**DOI:** 10.3389/fgene.2013.00049

**Published:** 2013-04-05

**Authors:** Xianyong Lan, Evan C. Cretney, Jenna Kropp, Karam Khateeb, Mary A. Berg, Francisco Peñagaricano, Ronald Magness, Amy E. Radunz, Hasan Khatib

**Affiliations:** ^1^College of Animal Science and Technology, Northwest Agriculture and Forestry UniversityYangling, China; ^2^Department of Animal Sciences, University of Wisconsin-MadisonMadison, WI, USA; ^3^Department of Obstetrics and Gynecology, University of Wisconsin-MadisonMadison, WI, USA

**Keywords:** maternal nutrition, pregnancy, gene expression, DNA methylation

## Abstract

Studies in rats and mice have established that maternal nutrition induces epigenetic modifications, sometimes permanently, that alter gene expression in the fetus, which in turn leads to phenotypic changes. However, limited data is available on the influence of maternal diet on epigenetic modifications and gene expression in sheep. Therefore, the objectives of this study were to investigate the impact of different maternal dietary energy sources on the expression of imprinted genes in fetuses in sheep. Ewes were naturally bred to a single sire and from days 67 ± 3 of gestation until necropsy (days 130 ± 1), they were fed one of three diets of alfalfa haylage (HY; fiber), corn (CN; starch), or dried corn distiller’s grains (DG; fiber plus protein plus fat). A total of 26 fetuses were removed from the dams and longissimus dorsi, semitendinosus, perirenal adipose depot, and subcutaneous adipose depot tissues were collected for expression and DNA methylation analyses. Expression analysis of nine imprinted genes and three DNA methyltransferase (DNMTs) genes showed significant effects of the different maternal diets on the expression of these genes. The methylation levels of CpG islands of both *IGF2R* and *H19* were higher in HY and DG than CN fetuses in both males and females. This result is consistent with the low amino acid content of the CN diet, a source of methyl group donors, compared to HY and DG diets. Thus, results of this study provide evidence of association between maternal nutrition during pregnancy and transcriptomic and epigenomic alterations of imprinted genes and DNMTs in the fetal tissues.

## Introduction

There is growing evidence that maternal diet during the different stages of pregnancy can induce physiological and epigenetic changes in fetal tissues in different species, which in turn could have serious implications after birth. Studies of the effects of undernutrition during gestation on babies conceived during the Dutch famine (1944–1945) showed that nutrient restriction can permanently impact adult health (Roseboom et al., 2001). Kaminen-Ahola et al. (2010) reported that exposure of pregnant mice to alcohol before or after fertilization affects gene expression in the offspring and can also impact postnatal body weight and skull size. Similarly, studies in sheep and cattle have shown that late-gestation maternal nutrition can impact postnatal progeny body composition, insulin sensitivity, and growth rate. For example, Micke et al. (2011) reported that growth of muscle tissues in bovine male calves was induced by consumption of a low-protein diet by their dams during the first and second trimester, which was also associated with increased expression of insulin-like growth factor 2 (*IGF2*). Recent work in early-weaned Angus calves reported that high-starch diets resulted in greater feed efficiency and lower residual feed intake and was associated with changes in gene expression and pathways involved in intramuscular adipocyte proliferation and differentiation (Graugnard et al., 2009, 2010). In sheep, increased nutrition during late pregnancy can lead to overexpression of genes regulating adipogenesis and lipogenesis in fetal perirenal fat tissue in sheep, which may result in increased adiposity in adult life (Muhlhausler et al., 2007).

Interestingly, the nutrition of the pregnant mother can modify epigenetic marks, such as DNA methylation, which in turn alters gene expression in the offspring without changing the DNA sequence. One of the best studied examples of epigenetically sensitive genes via maternal diet is the Agouti viable yellow (*A^vy^*) locus in the mouse. Methyl supplements in the diet of pregnant mice increased the methylation level of the Agouti gene, and consequently led to changes in the coat color (from brown to yellow) of the offspring and made them obese (Cooney et al., 2001; Waterland et al., 2008). These studies have clearly established that maternal diet has a transgenerational effect on the offspring through epigenetic modifications.

Epigenetic modifications, such as DNA methylation and acetylation, regulate expression of imprinted genes in a parent-of-origin manner so that alleles inherited from one parent are expressed, whereas alleles inherited from the other parent are silenced (Surani et al., 1984; Reik and Walter, 1998). To explain the evolution of genomic imprinting, Moore and Haig (1991) hypothesized that paternally expressed genes have evolved to promote growth of the fetus, whereas maternally expressed genes act to suppress growth of the fetus in order to allocate the mother’s resources for future pregnancies. Therefore, it is expected that imprinted genes would play a significant role in fetal growth and development. Indeed, it is evident that these genes have roles in embryonic survival and growth, obesity and insulin resistance, spermatogenesis defects, energy regulation, motor and learning dysfunction, deafness, weight loss, placental growth, tumorigenesis, apoptosis, fertility, and maternal behavior (http://www.mgu.har.mrc.ac.uk). Until recently, most studies of imprinted genes were focused on mouse and human. Nonetheless, there is an increasing body of knowledge on the roles of imprinted genes in livestock production. For example, in a recent genome scan, Imumorin et al. (2011) detected quantitative trait loci (QTL) with parent-of-origin effects on growth and carcass traits in the Angus × Brahman cattle crossbred population. A single nucleotide polymorphism (SNP) in *IGF2* was found to be associated with an increase in rib eye area and percentage of grade fat (Goodall and Schmutz, 2007). Magee et al. (2010) reported that SNPs in the bovine imprinted genes *CALCR*, *GRB10*, *PEG3*, *RASGRF1*, *ZIM2*, and *ZNF215* displayed associations with milk production, health, body condition, reproduction, carcass traits, and calf mortality.

Although expression of imprinted genes was found to be influenced by maternal nutrition in mouse and rat (Gong et al., 2010), the question of whether the expression of these genes in sheep fetuses is influenced by maternal diet has not been investigated. In addition, the enzymes known as DNA methyltransferases (*DNMT1*, *DNAMT3a*, and *DNMT3b*) catalyze the DNA methylation that occurs at cytosine bases, where *DNMT3a*, and *DNMT3b* establish these methylations and *DNMT1* maintains DNA methylation in the newly synthesized DNA strands (Denis et al., 2011). Previous studies in rats have reported altered expression of DNMTs in offspring as a result of different maternal diets (Lillycrop et al., 2007; Gong et al., 2010). Given the role of imprinted genes in fetal development and the sensitivity of pregnant dams to environmental factors such as diet, we hypothesize that maternal nutrition during pregnancy alters expression levels of imprinted genes and DNMTs in fetal and offspring tissues via epigenetic modifications, which in turn would lead to changes in fetal and adult programing. In this study, we report the impact of maternal dietary energy source during mid- to late-gestation on expression of imprinted genes and DNMTs in placental and progeny fetal tissues in sheep.

## Materials and Methods

### Ethics statement

The College of Agriculture and Life Sciences and the School of Medicine Animal Care and Use Committees of the University of Wisconsin-Madison approved the procedures used in this study.

### Animals, experimental design, and nutrition

Mature pregnant multiparous Polypay ewes (*n* = 15) were used in a randomized complete design to evaluate the effects of maternal dietary energy source during mid- to late-gestation on gene expression in maternal placenta, fetal muscle, and fetal adipose tissues. Ewes were all naturally bred to a single sire. From days 67 ± 3 of gestation until necropsy (days 130 ± 1), ewes were individually fed one of three primary energy sources initially formulated to contain 3.52 Mcal ME/day. The primary energy sources were from alfalfa haylage (HY; fiber), corn (CN; starch), or dried corn distiller’s grains (DG; protein, fat, fiber) (Table 1). Intake of CN and DG was limited to achieve isoenergetic intake among dietary treatments relative to an *ad libitum* intake of HY, and intake of CN and DG was adjusted to achieve similar body weight gains of ewes from days 67 to 130 of gestation (Radunz et al., 2011a). A more detailed description of diets and management is provided in Radunz et al. (2011a).

**Table 1:** **Daily nutrient intake of dams from days 67 to 130 of gestation in sheep**.

	Treatment		
Item	HY	CN	DG
DMI, kg/day	2.03	1.17	1.18
Alfalfa haylage	2.03	0.14	0.17
Corn	–	0.80	–
DDGS	–	–	0.77
Supplement	–	0.23	0.24
*Analyzed nutrient intake*			
Crude protein (g/days)	383.26	130.63	309.84
NDF (g/days)	940.10	198.82	508.16
Crude fat (g/days)	85.97	84.94	114.02
Methionine (g/days)	6.22	1.47	4.68
Cystine (g/days)	3.96	1.31	2.80
Serine (g/days)	57.9	8.55	15.8
Choline (mg/days)	29.3	7.2	24.0
Folate (mg/days)	0.05	6.5	11.4

*Treatment diets fed to dams: HY, *ad libitum* fed alfalfa haylage; CN, limit-fed whole shell corn; DG, limit-fed corn dried distillers grains; DMI, dry matter intake*.

### Tissue sample collection

The dams were necropsied on days 130 ± 1 of gestation. A total of 14 placental tissues were isolated from dams and cotyledon “A” type were used in this experiment. Additionally, a total of 26 fetuses were removed from the dams and four different tissue samples were collected from each of these fetuses. Longissimus dorsi and semitendinosus muscles were removed from the left side of the fetus and a muscle tissue sample was then collected. Adipose tissue samples were obtained from perirenal adipose depot and subcutaneous adipose depot near the shoulder blade. All tissues were frozen immediately at −80°C until RNA extraction was performed.

#### Total RNA extraction and reverse transcription

Total RNA was extracted from tissue samples (approximately 30 mg) using the RNeasy Mini kit (QIAGEN, Valencia, CA, USA) and treated with DNase-free DNase Set (QIAGEN) Concentrations and OD_260/280_ ratios of RNA samples were measured with the Nanodrop ND-1000 spectrophotometer (Nanodrop Technologies, Montchanin, DE, USA). A total of 1 μg RNA from each sample was used to synthesize cDNA using the iScript cDNA Synthesis Kit (Bio-Rad Laboratories, Hercules, CA, USA) following the manufacturer’s instructions.

#### Gene selection and quantitative real-time PCR

Given the roles of imprinted genes and DNMTs in the regulation of pre-natal growth, development, fertility, and production, four maternally expressed genes (*H19*, *IGF2R*, *GRB10*, and *MEG8*) (Thurston et al., 2008; Fleming-Waddell et al., 2009), five reported paternally expressed genes (*IGF2*, *PEG1*, *PEG3*, *DLK1*, and *DIO3*) (Thurston et al., 2008; Colosimo et al., 2009; Zhao et al., 2012), and three DNMTs (*DNMT1*, *DNMT3a*, and *DNMT3b*) were chosen for expression analysis in the fetal sheep tissues. The genes peroxisome proliferator-activated receptor gamma (*PPARG*) and CCAAT/enhancer binding protein (C/EBP), alpha (*CEBPA*) were also chosen in sheep because of their roles in the transcriptional network controlling adipogenesis.

Primers (Table 2) were designed to amplify fragments spanning more than one exon to exclude the possibility of genomic DNA contamination in the qPCR reactions using the Beacon Designer software 7.0 (Premier Biosoft International, Palo Alto, CA, USA). Imprinted genes, *PPARG*, and *CEBPA* were amplified in each tissue for each individual as described above. For expression analysis of DNMTs, two biological replicates of RNA pools from males and two from females were constructed and hence a total of 12 RNA pools were constructed from each tissue for the three maternal diets. All samples were run in triplicates in the ECO real-time PCR system (Illumina, San Diego, CA, USA) using the iQ™ SYBR Green Supermix kit (Bio-Rad Laboratories). The cycling conditions were 50°C for 2 min, 95°C for 10 min, and 40 cycles at 95°C for 10 s and 60°C for 30 s.

**Table 2:** **Primers used for relative gene expression using quantitative real-time-PCR**.

Gene	Forward primer	Reverse primer	Product Size (bp)	Reference
*H19*	CTCTTCAGACACCCAGAA	AAGTCCAAGTTCCAAGTC	79	–
*IGF2R*	ATGACTTGAAGACAGACA	ATAAGAAGGTGATGCTACT	138	–
*GRB10*	CACACCTGGGATTAGAGC	TGGGAAGAAATTCATGGGATT	143	–
*MEG8*	CCCAGGGAGTGTGAGGCTCTTCT	GGACCCACGGCTGACCTGTT	100	Bidwell et al. (2004)
*IGF2*	ATGTGTCTGCCTCTACGA	AGGTGTCAGATTGGAAGAAC	78	–
*PEG1.2*	ATCTGGTCGGCTTACAATCA	AAGGAGAGGACGGTGAGT	76	–
*PEG3*	TCTACGACTTCAGAGAGG	GCTCTTCCGAGAATGAAT	83	–
*DLK1*	CCCGTCCTCTTGCTCCTGCT	GGCTGGCACCTGCACACACT	116	Bidwell et al. (2004)
*DIO3*	CGACTTCCTCATCATCTAC	ATGCTGTAGGGAGAGTCT	75	–
*PPARG*	TAGGTGTGATCTTAACTGT	CTGATGGCATTATGAGAC	105	–
*CEBPA*	GGAGACGCAGCAGAAGGT	CGGCTCAGTTGTTCCACC	75	–
*DNMT1*	CCTGCTTCAGCGTGTACTGT	ATCGGCTTTGCTGAACCAGA	103	–
*DNMT3a*	TGTACGAGGTACGGCAGAAGTG	GGCTCCCACAAGAGATGCA	60	Grazul-Bilska et al. (2011)
*DNMT3b*	AGCGGCAGGCGATGTCT	GAGAACTTGCCATCACCAAACC	57	Grazul-Bilska et al. (2011)
*RPL19*	CAACTCCCGCCAGCAGAT	CCGGGAATGGACAGTCACA	76	Garcia-Crespo et al. (2005)
*GAPDH*	CCTGCCAAGTATGATGAGAT	TGAGTGTCGCTGTTGAAGT	119	Bidwell et al. (2004)
*ACTB*	CTGAGCGCAAGTACTCCGTGT	GCATTTGCGGTGGACGAT	125	Garcia-Crespo et al. (2005)

The internal control gene selection for normalization was as described in Vandesompele et al. (2002). The control gene producing the smallest relative stability value *M* (the average of the pair-wise variation when compared with the other control genes) across the examined samples was chosen for normalization. The housekeeping genes beta-actin (*ACTB*), glyceraldehyde-3-phosphate dehydrogenase (*GAPDH*), and ribosomal protein L19 (*PRL19*) were tested across tissue samples. *PRL19* was chosen as the internal control for placenta (*M* = 0.070), subcutaneous adipose (*M* = 0.046), longissimus dorsi (*M* = 0.013), and semitendinosus muscle (*M* = 0.014) tissues, and *ACTB* was selected as an internal control for perirenal fat tissue (*M* = 0.1482).

### Gene expression analysis

Normalized gene expression values (ΔCt) were analyzed using a general linear model. The nine imprinted genes, *PPARG*, and *CEBPA* were analyzed in sheep fetal tissues as follows: for sheep placenta, the statistical model included the fixed effect of the treatment (HY, CN, or DG) and the fixed effect of the type of the pregnancy (with two levels: single fetus or multiple fetuses) and for the other four sheep tissues, the model included the fixed effect of the treatment (HY, CN, or DG), the fixed effect of the sex of the fetus (male or female), and the random effect of the dam. Gene expression of DNMTs was analyzed using RNA pools and hence the statistical model included only treatment (for placenta) or treatment and sex as fixed effects (for fetal tissues). Association between the normalized gene expression and the treatment was tested using a likelihood ratio test by comparing this model to a reduced model without the treatment effect. The mean and the range of the fold change for each gene were calculated as 2^−ΔΔCt^ using the estimated ΔΔCt value ± standard error (Livak and Schmittgen, 2001). All analyses were performed using the lme4 package of R language/environment (R Development Core Team, 2009).

### Analysis of DNA methylation of *IGF2R* and *H19* by bisulfite sequencing

The IGF2R and H19 genes showed significant differential expression between the diet groups in longissimus dorsi and semitendinosus fetal muscle tissues in sheep. To evaluate the methylation status of *IGF2R* and *H19*, genomic DNA was isolated from longissimus dorsi and semitendinosus muscle tissues of 26 fetuses using a standard salt-chloroform extraction protocol (Müllenbach et al., 1989). Six DNA pools of fetal DNA tissues were constructed based on maternal diet and sex of the fetus: CN-F (females from corn-fed mothers; *n* = 3); CN-M (males from corn-fed mothers; *n* = 6); DG-F (*n* = 4); DG-M (*n* = 4); HY-F (*n* = 4); and HY-M (*n* = 5). DNA pools contained equal amounts of DNA from each individual. DNA pools (1 μg each) were treated with bisulfite and purified using the Epitect Bisulfite Kit (Qiagen). DNA pools were then amplified by a PCR reaction using the primers DMR-2-F AGGGGGTAGGAGTTAGAGTAGA and DMR-2-R ACCTTCTCAACACCTTACTCAAA for *IGF2R* and the primers H19-F ATT TTA AAT AGG GTT GAG AGG TTG T and H19-R AAA CAC AAA AAA TCC CTC ATT ATC for *H19* (Thurston et al., 2008). These primers amplify the differentially methylated region 2 (DMR2) of *IGF2R*, which is located in intron 2, and of *H19*, respectively. The PCR products were gel purified, ligated to the pGEM-T Vector (Promega, Madison, WI, USA), and transformed into JM101 competent cells (Promega) following the manufacturer’s instructions. Bacterial colonies were screened for the presence of a single copy of insert fragment by PCR with primers T7 and SP6 of the vector. For each sample, an average of 50 clones was sequenced to obtain bisulfite-converted DNA sequences. For each gene, the number of methylated and unmethylated CpGs sites was counted in each sample. Association of DNA methylation with maternal diet and sex was then tested using Fisher’s exact test.

## Results and Discussion

The expression profiles of nine imprinted, two adipogenic, and three DNMTs genes were estimated in four sheep fetal tissues from 26 fetuses belonging to three maternal dietary treatment groups (HY, CN, DG) and corresponding dam placental tissues using quantitative real-time PCR (qRT-PCR). Table 3 shows the *P*-values of the effects of the maternal diets and sex of the fetus on the expression levels of the examined genes in the four fetal tissues. Neither diet nor birth type (single or multiple fetuses per pregnancy) showed any significant associations with expression level of the examined genes in placental tissues.

**Table 3:** ***P-*values of the effects of diet and sex on the expression of 11 genes in four fetal sheep tissues**.

Gene	Perirenal fat		Subcutaneous fat		Longissimus dorsi		Semitendinosus	
	Diet	Sex	Diet	Sex	Diet	Sex	Diet	Sex
*H19*	0.647	0.211	0.148	0.673	0.352	0.385	<0.001	0.946
*IGF2R*	0.050	0.488	0.864	0.187	0.014	0.661	0.128	0.748
*GRB10*	0.056	0.782	0.152	0.147	0.701	0.714	0.076	0.498
*MEG8*	0.720	0.149	0.587	0.179	0.408	0.896	0.015	0.226
*IGF2*	0.027	0.462	0.137	0.025	0.279	0.088	0.205	0.312
*PEG1.2*	0.533	0.641	0.497	0.974	0.189	0.211	<0.001	0.909
*PEG3*	0.395	0.244	0.599	0.608	0.567	0.705	0.14	0.711
*DLK1*	0.979	0.726	0.693	0.639	0.028	0.813	0.055	0.941
*DIO3*	0.464	0.078	0.16	0.45	0.087	0.063	0.305	0.386
*PPARG*	0.594	0.702	0.126	0.021	0.332	0.576	0.21	0.687
*CEBPA*	0.987	0.704	0.007	0.386	0.541	0.157	0.071	0.582

### Differential expression of *IGF2*, *IGF2R*, and *GRB10* in fetal perirenal fat

**Figure 1:**
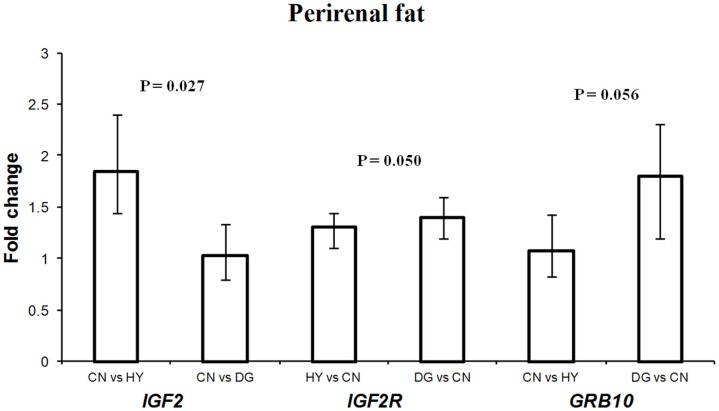
**Fold change differences in expression of *IGF2*, *IGF2R*, and *GRB10* in sheep fetal perirenal fat using quantitative real-time PCR (qRT-PCR)**. Data are shown as mean ± maximum- and minimum-fold changes between maternal diets. HY, *ad libitum* fed alfalfa haylage; CN, limit-fed whole shell corn; DG, limit-fed corn dried distillers grains.

Maternal dietary energy source showed significant effects on the expression of *IGF2* (*P* = 0.027), its receptor *IGF2R* (*P* = 0.050), and close to significant effects on the expression of *GRB10* (*P* = 0.056) (Table 3). *IGF2* showed 1.85-fold difference in expression in fetuses from dams fed CN vs. HY, whereas expression levels in fetuses of CN-fed and DG-fed dams were similar (Figure 1). *IGF2* is a paternally expressed gene and is located on ovine chromosome 21 (McLaren and Montgomery, 1999). Van Laere et al. (2003) identified a causative SNP in intron 3 of *IGF2* that affects muscle growth and fat deposition in pigs, which was consistent with the previous finding by Nezer et al. (1999). Also, associations between the causative SNP in *IGF2* and subcutaneous fat thickness and total body fat content have been reported in different pig breeds (Oczkowicz et al., 2009; Fontanesi et al., 2010; Burgos et al., 2012).

In contrast to *IGF2*, the expression of *IGF2R*, a maternally expressed gene, in perirenal fat was higher in fetuses from dams fed HY (1.3-fold difference) and DG (1.4-fold difference) than CN, and had similar expression levels in fetuses from dams fed HY and DG (Figure 1). In a previous study using the same dietary treatments, progeny from dams fed DG had greater internal (mostly perirenal) fat deposition than progeny from dams fed HY or CN (Radunz et al., 2011b). *IGF2* promotes fetal growth and development and cell survival and differentiation. In contrast, *IGF2R*, which is localized to the cell membrane, acts as a growth inhibitor by binding and internalizing *IGF2* into the cell for lysosomal degradation (Brown et al., 2009). Furthermore, *IGF2* stimulates the proliferation and differentiation of pre-adipocytes in brown adipose tissues through binding insulin receptor form A (IR-A) and the IGF-1 receptor (Viengchareun et al., 2008). Thus, the changes in expression levels of *IGF2* and *IGF2R* in fetal perirenal fat tissues as a response to maternal diet imply important roles of these genes in differentiation of adipose tissue. Also, the high expression of *IGF2* (paternally expressed) in fetuses whose dams were fed high-starch CN diet, which promotes fetal growth, provide further support to the parent-offspring conflict hypothesis proposed by Moore and Haig (1991).

*GRB10* was expressed greater in perirenal fat of fetuses from DG-fed mothers (1.8-fold difference) compared to CN-fed mothers and showed similar expression levels in fetuses from CN and HY-fed mothers (Figure 1). *GRB10*, a maternally expressed gene, is highly expressed in tissues targeted by insulin such as skeletal muscle and adipose tissue, although its role in the regulation of insulin signaling is not well-defined (Holt and Siddle, 2005). In our previous studies using a similar model, dams fed DG had greater circulating insulin during gestation compared to CN-fed and HY-fed counterparts (Radunz et al., 2011b). Charalambous et al. (2003) reported that aberrant expression of the maternal allele resulted in about 30% heavier mice than their wild type littermates, which suggests *GRB10* as an inhibitor of embryonic growth and development. Also, it has been reported that *GRB10* promotes adipogenesis (Smith et al., 2006). Perirenal fat tissues from fetuses whose mothers were fed high-starch CN diet, which has been reported to enhance fetal growth, displayed lower expression of *GRB10* compared to fetuses from dams fed HY or DG diets. Thus, these results further support the conflict hypothesis (Moore and Haig, 1991) that maternally expressed genes suppress fetal growth.

### Differential expression of *CEBPA*, *PPARG*, and *IGF2* in fetal subcutaneous adipose tissue

**Figure 2:**
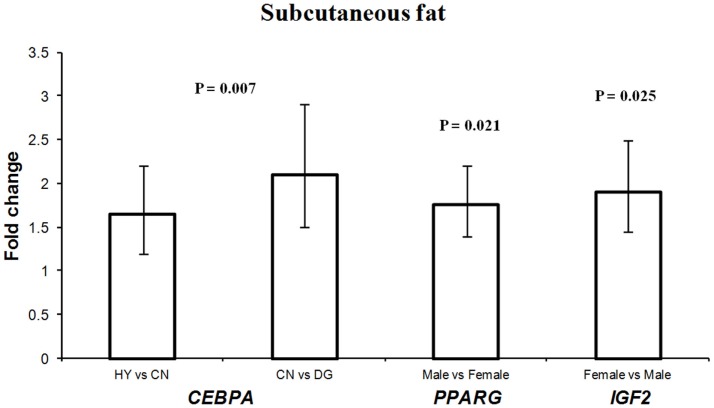
**Fold change differences in expression of *CEBPA*, *PPARG*, and *IGF2* in sheep fetal subcutaneous adipose tissue using quantitative real-time PCR (qRT-PCR)**. Data are shown as mean ± maximum- and minimum-fold changes between maternal diets and fetus sex. HY, *ad libitum* fed alfalfa haylage; CN, limit-fed whole shell corn; DG, limit-fed corn dried distillers grains.

The different maternal diets showed significant effects on the expression of *CEBPA* (*P* = 0.007) in subcutaneous adipose tissue (Table 3). *CEBPA* had greater expression in fetuses from HY-fed mothers than in fetuses from CN and DG-fed mothers (1.65- and 3.4-fold difference, respectively) (Figure 2). Armengol et al. (2012) found that the transcription factor *Cebpa* activates *Dlk1* in brown adipocytes in mice by binding to its promoter and that knockdown of *Cebpa* resulted in reduced expression of *Dlk1*. Given that *Dlk1* functions as a repressor of adipogenesis (Rosen and MacDougald, 2006), it can be concluded that *Cebpa* is a transcriptional regulator of *Dlk1* and has a key role in brown adipocyte differentiation in mice. Wang et al. (2012) reported that the expression of *CEBPA* in subcutaneous adipose tissue was the highest among 14 other Qinchuan cattle tissues. The function of *CEBPA* in sheep tissues is not clear. However, the differential expression of this gene in fetuses whose mothers were fed different energy sources suggests an important role in fetal programing of sheep subcutaneous adipose tissue.

Interestingly, *PPARG* showed 1.75-fold difference in expression in males vs. females, and *IGF2* showed 1.9-fold difference in females vs. males in subcutaneous adipose tissue (Figure 2). Recently, it has been reported that high-fat diet during late pregnancy resulted in differential expression and differential DNA methylation of imprinted genes in placenta from male and female mice (Gallou-Kabani et al., 2010). Also, a reduced expression of the rat *H19* and *IGF2* genes was observed in fetal liver in response to maternal low-protein diet during pre-implantation (Kwong et al., 2006). These sex-specific expression differences may lead to changes in postnatal growth and development (Kwong et al., 2006).

### Differential expression of *DLK1*, *IGF2R*, *DIO3*, *H19*, *PEG1*, and *MEG8* in fetal muscle tissues

**Figure 3:**
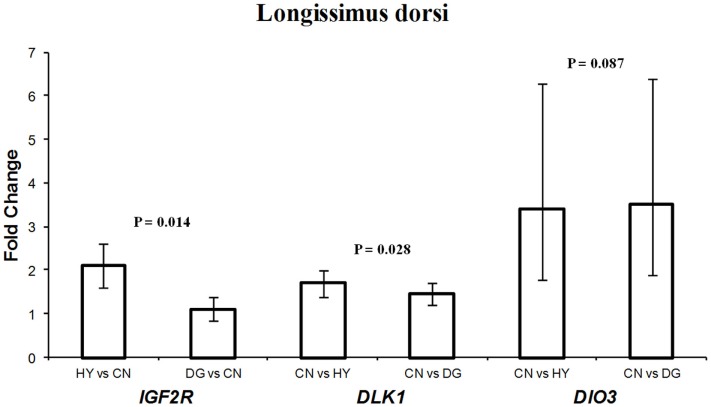
**Fold change differences in expression of *IGF2R*, *DLK1*, and *DIO3* in sheep fetal longissimus dorsi muscle using quantitative real-time PCR (qRT-PCR)**. Data are shown as mean ± maximum- and minimum-fold changes between maternal diets. HY, *ad libitum* fed alfalfa haylage; CN, limit-fed whole shell corn; DG, limit-fed corn dried distillers grains.

For longissimus dorsi muscle, expression levels of *DLK1* and *IGF2R* in the fetuses were affected significantly by the dietary energy source of the dams (Table 3). Fetuses from HY-fed dams had 2.1-fold higher expression difference of *IGF2R* compared to fetuses from CN-fed dams, whereas CN and DG fetuses showed similar expression levels of *IGF2R* (Figure 3). In contrast, *DLK1* had greater expression (*P* = 0.028) in fetuses from CN-fed dams than that of HY- and DG-fed dams (Figure 3). *DIO3* showed greater expression levels in fetuses from dams fed CN compared to HY, although it did not reach the statistical significance level (*P* = 0.087). The genes *H19* (*P* < 0.001), *MEG8* (*P* = 0.015), *PEG1* (*P* < 0.001), and *DLK1* (*P* = 0.055) showed remarkable expression differences in fetal semitendinosus muscle from dams fed different dietary energy sources during gestation (Table 3).

**Figure 4:**
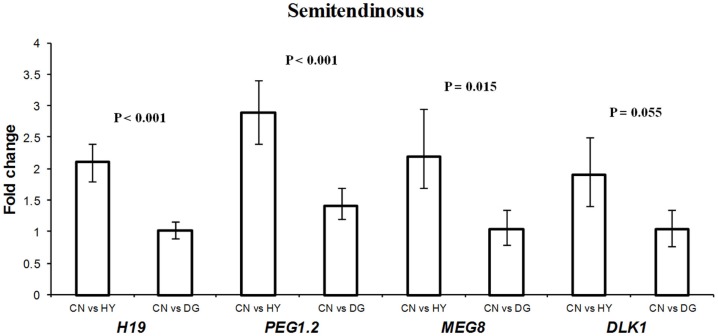
**Fold change differences in expression of *H19*, *PEG1*.2, *MEG8*, and *DLK1* in sheep fetal semitendinosus muscle using quantitative real-time PCR (qRT-PCR)**. Data are shown as mean ± maximum- and minimum-fold changes between maternal diets. HY, *ad libitum* fed alfalfa haylage; CN, limit-fed whole shell corn; DG, limit-fed corn dried distillers grains.

*DLK1* had greater expression in longissimus dorsi and semitendinosus muscle tissues from fetuses from dams fed high-starch (CN) vs. low-starch (HY) (Figures 3,4). The functions of *DLK1* in muscle and fat tissues from different species have been well-established. Moon et al. (2002) reported that *Dlk1* knockout mice display obesity and skeletal defects among other malformations. Also, the authors observed that mice lacking *Dlk1* had a significant increase in white adipose tissue accumulation when fed a high-fat diet compared to normal diet, which suggests that *Dlk1* has some functions in adaptation to positive energy balance (Moon et al., 2002). Furthermore, it has been suggested that overexpression of this gene in skeletal muscle leads to the muscular hypertrophy observed in callipyge sheep (Davis et al., 2004). Given that *DLK1* is an inhibitor of adipogenesis, as it prevents the differentiation of pre-adipocytes into mature adipocytes (Wang and Sul, 2006), it is possible that the elevated expression observed in longissimus dorsi and semitendinosus tissues after maternal high-starch diet may play a role in shifting more energy to muscle growth instead of intramuscular adipose tissue deposition. In previous cattle study investigating similar maternal diets during late-gestation, progeny from dams fed CN had less intramuscular fat deposition than progeny from dams fed HY (Radunz et al., 2012).

The expression pattern of *IGF2R* in longissimus dorsi (Figure 4) was consistent with that in perirenal fat (Figure 1). In both tissues, the gene was expressed less in fetuses from CN-fed mothers, a diet that may provide the fetus with more available glucose for growth. In previous cattle and sheep studies, birth weight of progeny from CN-fed dams was greater than progeny from HY-fed dams (Radunz et al., 2011a, 2012). Given that *IGF2R* is involved in the degradation of *IGF2*, a growth-promoting factor, our results suggest that *IGF2R* of the fetus responds to maternal nutrients by adapting expression levels that maintain its function as a growth suppressor.

*DIO3* is imprinted in mouse and encodes Type 3 iodothyronine deiodine (D3) enzyme, which degrades thyroid hormone when elevated in placenta and uterus. Although the imprinting status in sheep is not known, *DIO3* was included in this study because of its location in the highly conserved *DLK1*-*DIO3* region across mammalian species (Charlier et al., 2001). The function of *DIO3* in sheep is not known. However, mice lacking this gene show accumulation of thyroid hormone at birth and hypothyroidism in adulthood, which suggests that *DIO3* has important roles in developmental programing (da Rocha et al., 2008). Also, it has been suggested that alterations in expression of the *DLK1*-*DIO3* genes lead to growth and developmental deficiencies (da Rocha et al., 2008). Thus, it is conceivable to assume that the high-starch diet (CN) induces changes in longissimus muscle that are mediated by the elevation of *DIO3* expression.

*H19* showed 2.1-fold higher expression in the fetuses from dams fed CN compared to fetuses from dams fed HY (*P* < 0.001), with similar expression levels in fetuses from dams fed CN and DG (*P* > 0.05) (Figure 4). The biological function of *H19* has remained unclear since its discovery in 1984. *H19* was reported to be highly expressed in fetal organs and repressed after birth in the mouse (Pachnis et al., 1984) and human (Goshen et al., 1993; Ariel et al., 1997), which suggests important roles in embryogenesis and fetal growth. Indeed, *H19* knockout in mice resulted in overexpression of the growth factor *IGF2*, which in turn lead to overgrowth (Smith et al., 2006). In contrast to human and mouse, *H19* was expressed in skeletal muscle from lambs and adult sheep (Lee et al., 2002) and adult muscle tissues from cattle (Khatib and Schutzkus, 2006). Thus, the elevated expression of *H19* in semitendinosus muscle as a response to the maternal CN diet implies growth-related functions of this gene in muscle.

The expression level of splice variant 2 of *PEG1* (*PEG1.2*) was significantly higher in CN than DG and HY fetuses (Figure 4). The exact function of *PEG1* is not known yet although some roles in adipogenesis have been suggested. For example, Takahashi et al. (2005) reported that overexpression of *Peg1* in transgenic mice adipose tissue leads to enhanced expression of adipogenic genes (e.g., *PPARG*) and to hypertrophy of adipocytes. Also, it has been reported that *PEG1* is involved in the inhibition of Wnt/β-catenin signaling, which is essential for proper adipogenic differentiation (Jung et al., 2011). Inactivation of *Peg1* mouse ES cells resulted in *Peg1*-deficient mothers with decreased size and abnormal maternal behavior such as no response to newborns and leaving them unattended (Lefebvre et al., 1998). The authors concluded that *Peg1* is a positive regulator of embryonic growth and development. Thus, the greater expression observed in fetal semitendinosus muscle as a response to maternal CN diet supports previous studies on the role of *PEG1* in fetal growth. The mechanisms by which maternal diet affects gene expression are not well understood. However, in a recent study it was shown that *Peg1* expression was upregulated in white adipose tissues from mice fed a high-fat diet for 16 weeks and that this upregulation is not regulated by DNA methylation (Okada et al., 2009).

The expression of *MEG8* in fetal semitendinosus muscle as a response to maternal CN diet was greater than that of maternal HY diet (Figure 4). Expression analysis of *MEG8* in different sheep tissues showed exclusive expression in muscle (Charlier et al., 2001) and analysis of predicted sequences and ESTs matching the gene revealed absence of an ORF, which suggests that the ovine *MEG8* is a non-coding gene (da Rocha et al., 2008). Furthermore, the location of this gene within the *DLK1*-*DIO3* imprinted domain, which has important roles in muscle differentiation and growth, suggests a regulatory role in the expression of genes in this domain (da Rocha et al., 2008; Gu et al., 2012). The function of *MEG8* is not known, however, the observed differential expression in this study as a result of the different maternal diets suggests functional roles in fetal muscle growth.

### DNA methylation of *IGF2R* and *H19* in longissimus dorsi and semitendinosus fetal muscle tissues

**Figure 5:**
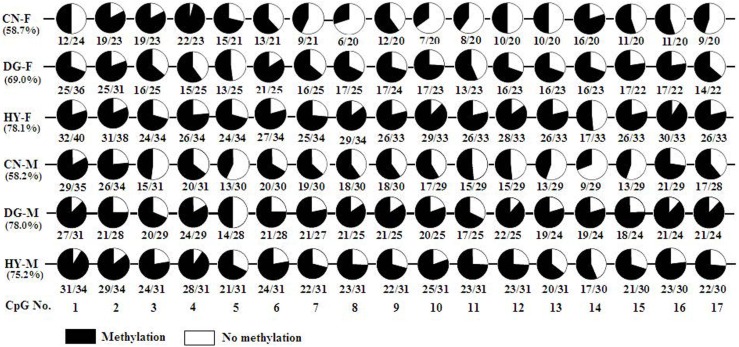
**DNA methylation of DMR2 region of *IGF2R* in sheep fetal longissimus dorsi muscle according to fetus sex (M, male; F, female) and maternal diet (HY, *ad libitum* fed alfalfa haylage; CN, limit-fed whole shell corn; DG, limit-fed corn dried distillers grains)**. Numbers below the circles indicate the portions of methylated CpGs versus unmethylated CpGs and percentages below diet types represent the percentage of methylated CpGs in each pool.

Expression of *IGF2R* was significantly higher in longissimus dorsi of fetuses from HY-fed mothers vs. fetuses from CN-fed mothers whereas expression of *H19* was found to be higher in CN compared to HY fetuses. DNA methylation is one of the most common mechanisms regulating transcription. Therefore, we sought to test whether or not DNA methylation of *IGF2R* and H19 is associated with the expression of the gene in male and female fetuses from dams that were fed different diets. DNA methylation analysis of DMR2 of *IGF2R*, which is located in intron 2 of the gene, revealed significant differences in methylation levels between the different maternal diets. Figure 5 shows that average DNA methylation of the 17 CpG sites of DMR2 was 0.587 in female fetuses from CN-fed mothers compared to 0.78 (*P* < 0.001) and 0.781 (*P* < 0.001) in DG and HY female fetuses, respectively. Similarly, the methylation level of DMR2 in longissimus dorsi of male fetuses from CN-fed mothers was 0.582 compared to 0.78 (*P* < 0.001) and 0.752 (*P* < 0.001) in DG and HY fetuses, respectively. Thus, methylation levels were consistently higher in HY and DG than CN fetuses in both males and females. The crude protein intake was remarkably higher in HY (383.26 g/day) and DG (309.84 g/days) diets compared to the CN diet (130.63 g/days). Specifically, the intake of amino acids methionine, cysteine, and serine were significantly higher in HY and DG diets than CN (Table 1). This suggests that the high methylation levels in HY and DG fetuses were probably due to the availability of amino acids in these high protein diets, which contributed methyl groups compared to the CN diet. Expression level of *IGF2R* also was significantly higher in HY than CN fetuses. Thus, a positive correlation between DNA methylation level and gene expression level was observed for *IGF2R* in longissimus muscle.

Until recently, most DNA methylation studies in mammals were focused on the promoter regions as methylation of these regions is used as a mechanism to suppress transcription and silence genes in the inactive X chromosome and imprinted genes (Jones and Takai, 2001). Thus, a negative correlation between DNA methylation in promoter regions and gene expression is well-established. Surprisingly, there is now accumulating evidence that intragenic methylation (gene body methylation) is abundant and that this methylation is positively correlated with gene expression (Hellman and Chess, 2007; Ball et al., 2009; Rauch et al., 2009; Aran et al., 2011; Jjingo et al., 2012). For example, Aran et al. (2011) reported positive correlation between gene body methylation levels and gene expression levels in B-lymphocyte cell lines, placenta, white blood cells, and fibroblasts, although this positive correlation was not a general characteristic of all human cells. Thus, the positive correlation between expression level of *IGF2R* and DNA methylation level in HY fetuses are consistent with these studies.

Although the mechanisms underlying this correlation are not clear, Jjingo et al. (2012) proposed a model which interprets the positive relationship between gene expression and gene body methylation. The authors suggested that at low levels of expression, the dense chromosome packaging prevents the access of DNMTs to DNA resulting in low DNA methylation. Also, at high expression levels, the high density of RNA Polymerase II interferes with DNMTs access to DNA. In contrast, at intermediate levels of expression, DNMTs have access to the DNA, which results in high gene body methylation (Jjingo et al., 2012). Consistent with this model, Suzuki and Bird (2008) suggested that gene body DNA methylation might be caused by anti-sense transcription in the active gene. Interestingly, *IGF2R* has an anti-sense non-coding RNA gene (*AIR*) that lies in the second intron of *IGF2R*, which includes the CpG island investigated in this study. Therefore, it is possible that the transcription of *AIR* allows access of both RNA Polymerase II and DNMTs to DNA which in turn results in both high DNA methylation and high expression of *IGF2R*.

Bisulfite sequencing of the 19 CpGs of *H19* DMR in semitendinosus muscle tissue revealed significant differential DNA methylation between the diet groups. DNA from CN fetuses showed 58.1% methylation level compared to 80.85% in the DG (*P* < 0.001) and 82.17% in the HY fetuses (*P* < 0.001). Furthermore, the expression of *H19* was significantly higher in the CN fetuses compared to the HY fetuses. The supplements of the HY diet to the dams during pregnancy included high content of amino acids that are donors of methyl groups (Table 1) compared to the CN diet, which could explain the elevated methylation level in the HY fetuses.

### Expression of DNMTS in fetal tissues

**Figure 6:**
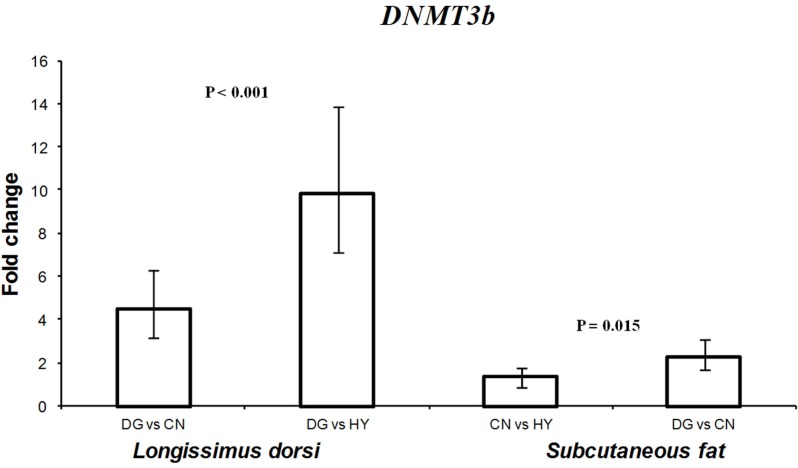
**Fold change differences in expression of *DNMT3b* in sheep fetal longissimus dorsi muscle and subcutaneous adipose tissue using quantitative real-time PCR (qRT-PCR)**. Data are shown as mean ± maximum- and minimum-fold changes between maternal diets. HY, *ad libitum* fed alfalfa haylage; CN, limit-fed whole shell corn; DG, limit-fed corn dried distillers grains.

Diet can alter DNA methylation by changing the concentration of *S*-adenosine methionine by the amount of methyl donors present in the diet (Zeisel, 2009; Dominguez-Salas et al., 2012). DNMTs genes play an important role in transferring methyl groups from *S*-adenosine methionine to the cytosine residues of DNA (Denis et al., 2011). Given that expression of imprinted genes is regulated by DNA methylation and that some imprinted genes showed different expression profiles as a response to the different diets, we hypothesized that maternal diet can influence the activity of DNMTs in fetal tissues. Therefore, one objective of this study was to test the effects of maternal diet on the expression of *DNMT1*, *DNMT3a*, and *DNMT3b* in male and female fetal tissues in sheep. Expression of *DNMT1* was higher (1.5-fold difference) in perirenal fat tissue of fetuses of DG mothers compared with CN-fed mothers (*P* = 0.039). Also, expression of *DNMT1* in female perirenal fat was significantly higher (*P* < 0.001) than male tissues with 2.8-fold expression difference. In contrast, *DNMT1* showed higher expression in longissimus muscle of male tissues compared with females. Similarly, *DNMT3a* showed twofold difference in expression in longissimus muscle between males and females (*P* = 0.033). *DNMT3b* expression was significantly altered in longissimus muscle (*P* < 0.001) and subcutaneous adipose (*P* = 0.015) fetal tissues as a response to maternal diet during late pregnancy (Figure 6). In longissimus muscle, *DNMT3b* showed 4.5- and 9.8-fold expression differences in fetuses from DG-fed compared with CN-fed and HY-fed mothers, respectively. In subcutaneous adipose tissue, *DNMT3b* showed 2.3-fold difference in expression between DG and CN groups.

Thus, *DNMT1*, *DNMT3a*, and *DNMT3b* showed sex- and tissue-specific expression patterns in the examined fetal tissues. Also, the different expression of DNMTs is consistent with previous studies in rats and pigs and indicates direct effects of maternal diets on the activity of both imprinted genes and DNMTs in fetal tissues. In the study of Lillycrop et al. (2007), it was found that the expression of *Dnmt1* mRNA was 17% lower in offspring from mothers fed a protein restricted diet compared to the control diet. Furthermore, when folic acid was used to supplement low-protein dies fed to pregnant rats, the level of *Dnmt1* expression could be recovered, providing further evidence that the maternal diet has an effect on the expression of this gene (Lillycrop et al., 2007). More recently, Gong et al. (2010) reported that both the expression levels of *DNMT1* and *DNMT3a* were significantly increased when pregnant rats were fed a low-protein diet. Also, Altmann et al. (2012) reported influences of maternal diet during gestation on the expression of DNMTs in fetal liver and muscle tissues in pigs. The tissue- and sex-specific differential expression of DNMTs found in our study suggests that establishment of altered DNA methylation by DNMTs can influence the phenotypic outcome of the offspring (Altmann et al., 2012).

### Dosage-sensitive effects of imprinted genes

It is well-established that many imprinted genes are sensitive to deviations from normal expression levels and that changes in the dosage of these genes cause abnormal development and growth deficiencies. A study by Bobetsis et al. (2010) found that threefold or less change in expression of imprinted genes was associated with abnormal development of mouse placenta. Also, Tunster et al. (2010) reported that doubling the expression of *Phlda2* in mice resulted in significant reductions in birth weight and embryonic glycogen stores. As such, the relatively small fold expression changes observed in this study could lead to pronounced phenotypic change in the offspring lifetime.

### Paternal vs. maternal expression

Interestingly, all paternally expressed genes examined in this study (*IGF2*, *DLK1*, *DIO3*, and *PEG1*) were highly expressed across fetal tissues as a response to the high-starch diet (CN), which supports fetal growth compared to the low-starch diet (HY). These results are consistent with the conflict hypothesis (Moore and Haig, 1991) that paternally expressed genes work to promote growth of the fetus. The expression patterns of the maternally expressed genes *IGF2R* and *GRB10* also were consistent with the conflict hypothesis. These genes were found to be lowly expressed in fetal perirenal fat tissue as a response to a high-starch diet (*IGF2R* and *GRB10*) and in longissimus dorsi (*IGF2R*). However, the maternally expressed genes *H19* and *MEG8* showed high expression in semitendinosus muscle in response to a high-starch-diet. Thus, the expression pattern of some imprinted genes is sometimes not consistent with the parent-offspring conflict hypothesis probably because of the multiple functions of these genes in the different tissues (Smith et al., 2006).

## Conclusion

The hypothesis of this study was that maternal nutrition during pregnancy induces epigenetic changes in the fetus, which in turn lead to alternations in gene expression of imprinted genes and DNMTs. The response of imprinted genes and DNMTs to different maternal energy sources in fetal tissues observed in this study suggests important roles in the adaptation to energy balance (Davis et al., 2004; da Rocha et al., 2008). The fact that some imprinted genes have regulatory functions in both embryonic growth and metabolism suggests important roles in fetal programing of these genes (Smith et al., 2006). Indeed, it has been suggested that expression changes of imprinted genes and DNMTs in early life may affect metabolic processes in adult animals (Gong et al., 2010; Altmann et al., 2012).

Dams consuming the CN diet had a lower protein intake than dams consuming the DG and HY diets. The observation that DNA methylation levels of *IGF2R* and *H19* were higher in fetuses from HY-fed and DG-fed mothers than CN fetuses may provide a direct evidence of influence of high protein content on DNA methylation in the fetus. Furthermore, the coupling of gene expression with DNA methylation observed for the CpG island in intron 2 of *IGF2R* supports the findings of a positive correlation between gene body methylation and gene expression recently reported. Overall, results of this study associate maternal nutrition with transcriptomic and epigenomic alterations of imprinted genes and DNMTs in the fetus. Therefore, future studies should focus on the long-term effects of these epigenetic changes on production and reproduction traits in livestock.

## Conflict of Interest Statement

The authors declare that the research was conducted in the absence of any commercial or financial relationships that could be construed as a potential conflict of interest.

## References

[B1] Altmann S. Murani E. Schwerin M. Metges C. C. Wimmers K. Ponsuksili S. (2012). Maternal dietary protein restriction and excess affects offspring gene expression and methylation of non-SMC subunits of condensin I in liver and skeletal muscle. Epigenetics 7, 239–252 10.4161/epi.7.3.19183 22430800

[B2] Aran D. Toperoff G. Rosenberg M. Hellman A. (2011). Replication timing-related and gene body-specific methylation of active human genes. Hum. Mol. Genet. 20, 670–680 10.1093/hmg/ddq513 21112978

[B3] Ariel I. Ayesh S. Perlman E. J. Pizov G. Tanos V. Schneider T. (1997). The product of the imprinted H19 gene is an oncofetal RNA. Mol. Pathol. 50, 34–44 10.1136/mp.50.1.34 9208812 PMC379577

[B4] Armengol J. Villena J. A. Hondares E. Carmona M. C. Sul H. S. Iglesias R. (2012). Pref-1 in brown adipose tissue: specific involvement in brown adipocyte differentiation and regulatory role of C/EBPδ. Biochem. J. 443, 799–810 10.1042/BJ20111714 22324440

[B5] Ball M. P. Li J. B. Gao Y. Lee J. H. LeProust E. M. Park I. H. (2009). Targeted and genome-scale strategies reveal gene-body methylation signatures in human cells. Nat. Biotechnol. 27, 361–368 10.1038/nbt0509-485b 19329998 PMC3566772

[B6] Bidwell C. A. Kramer L. N. Perkins A. C. Hadfield T. S. Moody D. E. Cockett N. E. (2004). Expression of PEG11 and PEG11AS transcripts in normal and callipyge sheep. BMC Biol. 2:17 10.1186/1741-7007-2-17 15298706 PMC514575

[B7] Bobetsis Y. A. Barros S. P. Lin D. M. Arce R. M. Offenbacher S. (2010). Altered gene expression in murine placentas in an infection-induced intrauterine growth restriction model: a microarray analysis. J. Reprod. Immunol. 85, 140–148 10.1016/j.jri.2010.04.001 20478622 PMC2904600

[B8] Brown J. Jones E. Y. Forbes B. E. (2009). Keeping IGF-II under control: lessons from the IGF-II-IGF2R crystal structure. Trends Biochem. Sci. 34, 612–619 10.1016/j.tibs.2009.07.003 19796953

[B9] Burgos C. Galve A. Moreno C. Altarriba J. Reina R. García C. (2012). The effects of two alleles of IGF2 on fat content in pig carcasses and pork. Meat Sci. 90, 309–313 10.1016/j.meatsci.2011.07.016 21907500

[B10] Charalambous M. Smith F. M. Bennett W. R. Crew T. E. Mackenzie F. Ward A. (2003). Disruption of the imprinted Grb10 gene leads to disproportionate overgrowth by an Igf2-independent mechanism. Proc. Natl. Acad. Sci. U.S.A. 100, 8292–8297 10.1073/pnas.1532175100 12829789 PMC166222

[B11] Charlier C. Segers K. Wagenaar D. Karim L. Berghmans S. Jaillon O. (2001). Human-ovine comparative sequencing of a 250-kb imprinted domain encompassing the callipyge (clpg) locus and identification of six imprinted transcripts: DLK1. Genome Res. 11, 850–862 10.1101/gr.172701 11337479 PMC311092

[B12] Colosimo A. Rocco G. D. Curini V. Russo V. Capacchietti G. Berardinelli P. (2009). Characterization of the methylation status of five imprinted genes in sheep gametes. Anim. Genet. 40, 900–908 10.1111/j.1365-2052.2009.01939.x 19694650

[B13] Cooney C. A. Dave A. A. Wolff G. L. (2001). Maternal methyl supplements in mice affect epigenetic variation and DNA methylation of offspring. J. Nutr. 132(Suppl. 8), 2393S–2400S 12163699 10.1093/jn/132.8.2393S

[B14] da Rocha S. T. Edwards C. A. Ito M. Ogata T. Ferguson-Smith A. C. (2008). Genomic imprinting at the mammalian Dlk1-Dio3 domain. Trends Genet. 24, 306–316 10.1016/j.tig.2008.03.011 18471925

[B15] Davis E. Jensen C. H. Schroder H. D. Farnir F. Shay-Hadfield T. Kliem A. (2004). Ectopic expression of DLK1 protein in skeletal muscle of padumnal heterozygotes causes the callipyge phenotype. Curr. Biol. 14, 1858–1862 10.1016/j.cub.2004.09.079 15498495

[B16] Denis H. Ndlovu M. N. Fuks F. (2011). Regulation of mammalian DNA methyltransferases: a route to new mechanisms. EMBO Rep. 12, 647–656 10.1038/embor.2011.110 21660058 PMC3128952

[B17] Dominguez-Salas P. Cox S. E. Prentice A. M. Hennig B. J. Moore S. E. (2012). Maternal nutritional status, C-1 metabolism and offspring DNA methylation: a review of current evidence in human subjects. Proc. Nutr. Soc. 71, 154–165 10.1017/S0029665111003338 22124338 PMC3491641

[B18] Fleming-Waddell J. N. Olbricht G. R. Taxis T. M. White J. D. Vuocolo T. Craig B. A. (2009). Effect of DLK1 and RTL1 but not MEG3 or MEG8 on muscle gene expression in callipyge lambs. PLoS ONE 4:e7399 10.1371/journal.pone.0007399 19816583 PMC2756960

[B19] Fontanesi L. Speroni C. Buttazzoni L. Scotti E. Dall’Olio S. Nanni Costa L. (2010). The insulin-like growth factor 2 (IGF2) gene intron3-g.3072G>A polymorphism is not the only *Sus scrofa* chromosome 2p mutation affecting meat production and carcass traits in pigs: evidence from the effects of a cathepsin D (CTSD) gene polymorphism. J. Anim. Sci. 88, 2235–2245 10.2527/jas.2009-2560 20382874

[B20] Gallou-Kabani C. Gabory A. Tost J. Karimi M. Mayeur S. Lesage J. (2010). Sex- and diet-specific changes of imprinted gene expression and DNA methylation in mouse placenta under a high-fat diet. PLoS ONE 5:e14398 10.1371/journal.pone.0014398 21200436 PMC3006175

[B21] Garcia-Crespo D. Juste R. A. Hurtado A. (2005). Selection of ovine housekeeping genes for normalisation by real-time RT-PCR; analysis of PrP gene expression and genetic susceptibility to scrapie. BMC Vet. Res. 1:3 10.1186/1746-6148-1-3 16188044 PMC1262732

[B22] Gong L. Pan Y. X. Chen H. (2010). Gestational low protein diet in the rat mediates Igf2 gene expression in male offspring via altered hepatic DNA methylation. Epigenetics 5, 619–626 10.4161/epi.5.7.12882 20671425

[B23] Goodall J. J. Schmutz S. M. (2007). IGF2 gene characterization and association with rib eye area in beef cattle. Anim. Genet. 38, 154–161 10.1111/j.1365-2052.2007.01576.x 17403010

[B24] Goshen R. Rachmilewitz J. Schneider T. de-Groot N. Ariel I. Palti Z. (1993). The expression of the H-19 and IGF-2 genes during human embryogenesis and placental development. Mol. Reprod. Dev. 34, 374–379 10.1002/mrd.1080340405 7682421

[B25] Graugnard D. E. Berger L. L. Faulkner D. B. Loor J. J. (2010). High-starch diets induce precocious adipogenic gene network up-regulation in longissimus lumborum of early-weaned Angus cattle. Br. J. Nutr. 103, 953–963 10.1017/S0007114509992789 20021700

[B26] Graugnard D. E. Piantoni P. Bionaz M. Berger L. L. Faulkner D. B. Loor J. J. (2009). Adipogenic and energy metabolism gene networks in longissimus lumborum during rapid post-weaning growth in Angus and Angus x Simmental cattle fed high-starch or low-starch diets. BMC Genomics 10:142 10.1186/1471-2164-10-142 19335898 PMC2676302

[B27] Grazul-Bilska A. T. Johnson M. L. Borowicz P. P. Minten M. Bilski J. J. Wroblewski R. (2011). Placental development during early pregnancy in sheep: cell proliferation, global methylation, and angiogenesis in the fetal placenta. Reproduction 141, 529–540 10.1530/REP-10-0505 21273368

[B28] Gu T. He H. Han Z. Zeng T. Huang Z. Liu Q. (2012). Expression of macro non-coding RNAs Meg8 and Irm in mouse embryonic development. Acta Histochem. 114, 392–399 10.1016/j.acthis.2011.07.009 21855964

[B29] Hellman A. Chess A. (2007). Gene body-specific methylation on the active X chromosome. Science 315, 1141–1143 10.1126/science.1136352 17322062

[B30] Holt L. J. Siddle K. (2005). Grb10 and Grb14: enigmatic regulators of insulin action – and more? Biochem. J. 388(Pt 2), 393–406 10.1042/BJ20050216 15901248 PMC1138946

[B31] Imumorin I. G. Kim E. H. Lee Y. M. De Koning D. J. van Arendonk J. A. De Donato M. (2011). Genome scan for parent-of-origin QTL effects on bovine growth and carcass traits. Front. Genet. 2:44 10.3389/fgene.2011.00044 22303340 PMC3268597

[B32] Jjingo D. Conley A. B. Yi S. V. Lunyak V. V. Jordan I. K. (2012). On the presence and role of human gene-body DNA methylation. Oncotarget 3, 462–474 22577155 10.18632/oncotarget.497 PMC3380580

[B33] Jones P. A. Takai D. (2001). The role of DNA methylation in mammalian epigenetics. Science 293, 1068–1070 10.1126/science.1063852 11498573

[B34] Jung H. Lee S. K. Jho E. H. (2011). Mest/Peg1 inhibits Wnt signalling through regulation of LRP6 glycosylation. Biochem. J. 436, 263–269 10.1042/BJ20101512 21375506

[B35] Kaminen-Ahola N. Ahola A. Maga M. Mallitt K. A. Fahey P. Cox T. C. (2010). Maternal ethanol consumption alters the epigenotype and the phenotype of offspring in a mouse model. PLoS Genet. 6:e1000811 10.1371/journal.pgen.1000811 20084100 PMC2797299

[B36] Khatib H. Schutzkus V. (2006). The expression profile of the H19 gene in cattle. Mamm. Genome 17, 991–996 10.1007/s00335-006-0038-2 16964441

[B37] Kwong W. Y. Miller D. J. Ursell E. Wild A. E. Wilkins A. P. Osmond C. (2006). Imprinted gene expression in the rat embryo-fetal axis is altered in response to periconceptional maternal low protein diet. Reproduction 132, 265–277 10.1530/rep.1.01038 16885535

[B38] Lee R. S. Depree K. M. Davey H. W. (2002). The sheep (*Ovis aries*) H19 gene: genomic structure and expression patterns, from the preimplantation embryo to adulthood. Gene 301, 67–77 10.1016/S0378-1119(02)01085-5 12490325

[B39] Lefebvre L. Viville S. Barton S. C. Ishino F. Keverne E. B. Surani M. A. (1998). Abnormal maternal behaviour and growth retardation associated with loss of the imprinted gene Mest. Nat. Genet. 20, 163–169 10.1038/2464 9771709

[B40] Lillycrop K. A. Slater-Jefferies J. L. Hanson M. A. Godfrey K. M. Jackson A. A. Burdge G. C. (2007). Induction of altered epigenetic regulation of the hepatic glucocorticoid receptor in the offspring of rats fed a protein-restricted diet during pregnancy suggests that reduced DNA methyltransferase-1 expression is involved in impaired DNA methylation and changes in histone modifications. Br. J. Nutr. 97, 1064–1073 10.1017/S000711450769196X 17433129 PMC2211425

[B41] Livak K. J. Schmittgen T. D. (2001). Analysis of relative gene expression data using real-time quantitative PCR and the 2-ΔΔCT method. Methods 25, 402–408 10.1006/meth.2001.1262 11846609

[B42] Magee D. A. Sikora K. M. Berkowicz E. W. Berry D. P. Howard D. J. Mullen M. P. (2010). DNA sequence polymorphisms in a panel of eight candidate bovine imprinted genes and their association with performance traits in Irish Holstein-Friesian cattle. BMC Genet. 11:93 10.1186/1471-2350-11-93 20942903 PMC2965127

[B43] McLaren R. J. Montgomery G. W. (1999). Genomic imprinting of the insulin-like growth factor 2 gene in sheep. Mamm. Genome 10, 588–591 10.1007/s003359901050 10341091

[B44] Micke G. C. Sullivan T. M. McMillen I. C. Gentili S. Perry V. E. (2011). Protein intake duringgestation affects postnatal bovine skeletal muscle growth and relative expression of IGF1, IGF1R, IGF2 and IGF2R. Mol. Cell. Endocrinol. 332, 234–241 10.1016/j.mce.2010.10.018 21056085

[B45] Moon Y. S. Smas C. M. Lee K. Villena J. A. Kim K. H. Yun E. J. (2002). Mice lacking paternally expressed Pref-1/Dlk1 display growth retardation and accelerated adiposity. Mol. Cell. Biol. 22, 5585–5592 10.1128/MCB.22.15.5585-5592.2002 12101250 PMC133956

[B46] Moore T. Haig D. (1991). Genomic imprinting in mammalian development: a parental tug-of-war. Trends Genet. 7, 45–49 10.1016/0168-9525(91)90040-W 2035190

[B47] Muhlhausler B. S. Duffield J. A. McMillen I. C. (2007). Increased maternal nutrition stimulates peroxisome proliferator activated receptor-gamma, adiponectin, and leptin messenger ribonucleic acid expression in adipose tissue before birth. Endocrinology 148, 878–885 10.1210/en.2006-1115 17068138

[B48] Müllenbach R. Lagoda P. J. Welter C. (1989). An efficient salt-chloroform extraction of DNA from blood and tissue. Trends Genet. 5, 391 2623762

[B49] Nezer C. Moreau L. Brouwers B. Coppieters W. Detilleux J. Hanset R. (1999). An imprinted QTL with major effect on muscle mass and fat deposition maps to the IGF2 locus in pigs. Nat. Genet. 21, 155–156 10.1038/5935 9988262

[B50] Oczkowicz M. Tyra M. Walinowicz K. Rózycki M. Rejduch B. (2009). Known mutation (A3072G) in intron 3 of the IGF2 gene is associated with growth and carcass composition in Polish pig breeds. J. Appl. Genet. 50, 257–259 10.1007/BF03195681 19638682

[B51] Okada Y. Sakaue H. Nagare T. Kasuga M. (2009). Diet-induced up-regulation of gene expression in adipocytes without changes in DNA methylation. Kobe J. Med. Sci. 54, e241–249 19628964

[B52] Pachnis V. Belayew A. Tilghman S. M. (1984). Locus unlinked to alpha-fetoprotein under the control of the murine raf and Rif genes. Proc. Natl. Acad. Sci. U.S.A. 81, 5523–5527 10.1073/pnas.81.17.5523 6206499 PMC391738

[B53] R Development Core Team (2009). R: A Language and Environment for StatisticalComputing. Vienna: R Foundation for Statistical Computing

[B54] Radunz A. Fluharty F. Zerby H. Loerch S. (2011a). Winter-feeding systems for gestating sheep I. Effects on pre- and postpartum ewe performance and lamb progeny preweaning performance. J. Anim. Sci. 89, 467–477 10.2527/jas.2010-3037 21262977

[B55] Radunz A. E. Fluharty F. L. Susin I. Felix T. L. Zerby H. N. Loerch S. C. (2011b). Winter-feeding systems for gestating sheep II. Effects on feedlot performance, glucose tolerance, and carcass composition of lamb progeny. J. Anim. Sci. 89, 478–488 10.2527/jas.2010-3037 21262978

[B56] Radunz A. E. Fluharty F. L. Relling A. E. Felix T. L. Shoup L. M. Zerby H. N. (2012). Prepartum dietary energy source fed to beef cows: II. Effects on progeny postnatal growth, glucose tolerance, and carcass composition. J. Anim. Sci. 90, 4962–4974 10.2527/jas.2012-5098 22952375

[B57] Rauch T. A. Wu X. Zhong X. Riggs A. D. Pfeifer G. P. (2009). A human B cell methylome at 100-base pair resolution. Proc. Natl. Acad. Sci. U.S.A. 106, 671–678 10.1073/pnas.0812399106 19139413 PMC2621253

[B58] Reik W. Walter J. (1998). Imprinting mechanisms in mammals. Curr. Opin. Genet. Dev. 8, 154–164 10.1016/S0959-437X(98)80136-6 9610405

[B59] Roseboom T. J. van der Meulen J. H. Ravelli A. C. Osmond C. Barker D. J. Bleker O. P. (2001). Effects of prenatal exposure to the Dutch famine on adult disease in later life: an overview. Mol. Cell. Endocrinol. 185, 93–98 10.1016/S0303-7207(01)00721-3 11738798

[B60] Rosen E. D. MacDougald O. A. (2006). Adipocyte differentiation from the inside out. Nat. Rev. Mol. Cell Biol. 7, 885–896 10.1038/nrm2066 17139329

[B61] Smith F. M. Garfield A. S. Ward A. (2006). Regulation of growth and metabolism by imprinted genes. Cytogenet. Genome Res. 113, 279–291 10.1159/000090847 16575191

[B62] Surani M. A. Barton S. C. Norris M. L. (1984). Development of reconstituted mouse eggs suggests imprinting of the genome during gametogenesis. Nature 308, 548–550 10.1038/308548a0 6709062

[B63] Suzuki M. M. Bird A. (2008). DNA methylation landscapes: provocative insights from epigenomics. Nat. Rev. Genet. 9, 465–476 10.1038/nrg2341 18463664

[B64] Takahashi M. Kamei Y. Ezaki O. (2005). Mest/Peg1 imprinted gene enlarges adipocytes and is a marker of adipocyte size. Am. J. Physiol. Endocrinol. Metab. 288, e117–124 10.1152/ajpendo.00244.2004 15353408

[B65] Thurston A. Taylor J. Gardner J. Sinclair K. D. Young L. E. (2008). Monoallelic expression of nine imprinted genes in the sheep embryo occurs after the blastocyst stage. Reproduction 135, 29–40 10.1530/REP-07-0211 18159081

[B66] Tunster S. J. Tycko B. John R. M. (2010). The imprinted gene PHLDA2 regulates extraembryonic stores. Mol. Cell. Biol. 30, 295–306 10.1128/MCB.00662-09 19884348 PMC2798284

[B67] Van Laere A. S. Nguyen M. Braunschweig M. Nezer C. Collette C. Moreau L. (2003). A regulatory mutation in IGF2 causes a major QTL effect on muscle growth in the pig. Nature 425, 832–836 10.1038/nature02064 14574411

[B68] Vandesompele J. De Preter K. Pattyn F. Poppe B. Van Roy N. De Paepe A. (2002). Accurate normalization of real-time quantitative RT-PCR data by geometric averaging of multiple internal control genes. Genome Biol. 3, RESEARCH0034 10.1186/gb-2002-3-7-research0034 12184808 PMC126239

[B69] Viengchareun S. Servel N. Fève B. Freemark M. Lombès M. Binart N. (2008). Prolactin receptor signaling is essential for perinatal brown adipocyte function: a role for insulin-like growth factor-2. PLoS ONE 3:e1535 10.1371/journal.pone.0001535 18253483 PMC2212135

[B70] Wang H. Zan L. S. Wang H. B. Gong C. Fu C. Z. (2012). Cloning, expression analysis and sequence prediction of the CCAAT/enhancer-binding protein alpha gene of Qinchuan cattle. Genet. Mol. Res. 11, 1651–1661 10.4238/2012.February.16.3 22782584

[B71] Wang Y. Sul H. S. (2006). Ectodomain shedding of preadipocyte factor 1 (Pref-1) by tumor necrosis factor alpha converting enzyme (TACE) and inhibition of adipocyte differentiation. Mol. Cell. Biol. 26, 5421–5435 10.1128/MCB.02189-05 16809777 PMC1592724

[B72] Waterland R. A. Travisano M. Tahiliani K. G. Rached M. T. Mirza S. (2008). Methyl donor supplementation prevents transgenerational amplification of obesity. Int. J. Obes. (Lond.) 32, 1373–1379 10.1038/ijo.2008.100 18626486 PMC2574783

[B73] Zeisel S. H. (2009). Importance of methyl donors during reproduction. Am. J. Clin. Nutr. 89, 673S–677S 10.3945/ajcn.2008.26811F 19116320 PMC2628952

[B74] Zhao L. X. Zhao G. P. Guo R. Q. Zhang D. Li X. H. Zhou H. M. (2012). DNA methylation status in tissues of sheep clones. Reprod. Domest. Anim. 47, 504–512 10.1111/j.1439-0531.2011.01911.x 22039959

